# Insight Into Microbial Applications for the Biodegradation of Pyrethroid Insecticides

**DOI:** 10.3389/fmicb.2019.01778

**Published:** 2019-08-02

**Authors:** Pankaj Bhatt, Yaohua Huang, Hui Zhan, Shaohua Chen

**Affiliations:** State Key Laboratory for Conservation and Utilization of Subtropical Agro-Bioresources, Guangdong Province Key Laboratory of Microbial Signals and Disease Control, Integrative Microbiology Research Centre, South China Agricultural University, Guangzhou, China

**Keywords:** biodegradation, pyrethroids, metabolic pathway, esterase enzyme, hydrolysis

## Abstract

Pyrethroids are broad-spectrum insecticides and presence of chiral carbon differentiates among various forms of pyrethroids. Microbial approaches have emerged as a popular solution to counter pyrethroid toxicity to marine life and mammals. Bacterial and fungal strains can effectively degrade pyrethroids into non-toxic compounds. Different strains of bacteria and fungi such as *Bacillus* spp., *Raoultella ornithinolytica, Psudomonas flourescens*, *Brevibacterium* sp., *Acinetobactor* sp., *Aspergillus* sp., *Candida* sp., *Trichoderma* sp., and *Candia* spp., are used for the biodegradation of pyrethroids. Hydrolysis of ester bond by enzyme esterase/carboxyl esterase is the initial step in pyrethroid biodegradation. Esterase is found in bacteria, fungi, insect and mammalian liver microsome cells that indicates its hydrolysis ability in living cells. Biodegradation pattern and detected metabolites reveal microbial consumption of pyrethroids as carbon and nitrogen source. In this review, we aim to explore pyrethroid degrading strains, enzymes and metabolites produced by microbial strains. This review paper covers in-depth knowledge of pyrethroids and recommends possible solutions to minimize their environmental toxicity.

## Introduction

**Figure 1:**
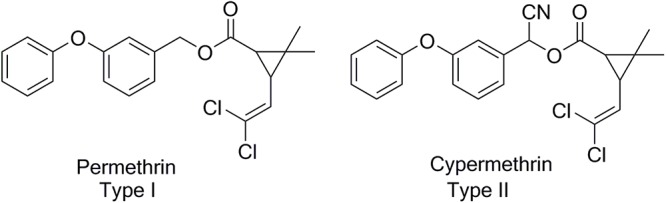
Structure of type I and type II pyrethroids.

Pyrethroids are the most commonly used global pesticides. *Chrysanthemum cinerariaefolium* flowers are the natural source of pyrethroids and allethrin was developed as the first synthetic pyrethroid insecticide in 1949 (Ensley, 2018; Gammon et al., 2019; Xu et al., 2019). Pyrethroids can be divided into two groups, type I pyrethroids containing basic cyclopropane carboxylic (e.g., allethrin) and type II pyrethroids containing cyano group (Proudfoot, 2005; Wolansky and Harrill, 2008; Chang et al., 2016; Figure 1). Presence of cyano group in type II pyrethroids enhances their insecticidal properties as compared to type I pyrethroids. All pyrethroids contain at least four stereoisomers, which exhibit different biological activities (Table 1). Pyrethroids are either marketed as racemic mixture of stereoisomers or single chemical isomer. Piperonyl butoxide acts as synergist in commercial formulation of pyrethroids and inhibits the metabolic degradation of active compounds (Bradberry et al., 2005; Fai et al., 2017). Deltamethrin is used in different countries to control malaria-spreading mosquitoes. Pyrethroids are reported to be 2250 times more toxic to insect than mammals and disrupt sodium, chloride channels (Chrustek et al., 2018). At high concentrations pyrethroids inhibit the functioning of gamma amino butyric acid (GABA) gated chloride ion channel (Bradberry et al., 2005; Gammon et al., 2019). Pyrethroids are mainly used to control insect pests of agriculture, horticulture, forestry and household. Pyrethroids are considered comparatively safe but their extensive use makes them harmful for humans and animals (Kuivila et al., 2012; Burns and Pastoor, 2018; Bordoni et al., 2019). Previous reports have concluded their detrimental effects on non-target species including marine fish and aquatic insects (Burns and Pastoor, 2018; Lu et al., 2019). Pyrethroid toxicity biomarkers have been well documented in fish (Ullah et al., 2019). Frequent pyrethroids applications in agriculture and households can cause inappropriate effects on human growth. In humans, pyrethroids exposure leads to contaminated urine, low serum quality, and antiandrogenic activity. Bio-absorption of pyrethroids was detected in the urine samples of outdoor workers in California (Sullivan et al., 2019), which indicates the importance of this topic. In rats, the developmental of bifenthrin neurotoxicity was reported as mixed type (typeI/II) (Gammon et al., 2019) whereas non-target neurotoxicity of pyrethroids has also been investigated in zebrafish (Paravani et al., 2017; Awoyemi et al., 2019; Strungaru et al., 2019).

**Table 1:** Brief classification of indoor and agricultural pyrethroids.

	Type I	Type II	Racemic pyrethroids
S.No.	pyrethroids	pyrethroids	(formulations with isomer)
1	Allethrin	Cyhalothrin	Resmethrin (bioresmethrin and *cis*-resmethrin
2	Bioallethrin	Cyfluthrin	Allethrin (d-allethrin, bioallethrin, esbiothrin, and s-bioallethrin)
3	Bifenthrin	γ-Cyhalothrin	Fenvalerate (esfenvalerate)
4	Permethrin	Cypermethrin	Cyhalothrin (γ-cyhalothrin)
5	D-Phenothrin	α-Cypermethrin	Phenothrin (d-phenothrin)
6	Prallethrin	Deltamethrin	Cypermethrin (d-cypermethrin)
7	Resmethrin	Fenpropathrin	
8	Bioresmethrin	Fenvalerate	
9	Tefluthrin	Esfenvalerate	
10	Tetramethrin	Flucythrinate	
11		Flumethrin	
12		Tau-fluvalinate	

Microbial system is considered suitable for the biodegradation of synthetic pyrethroids (Bhatt et al., 2019a). Most of the previous work about pyrethroids is based on bacterial degradation. Bacterial strains from the genera *Bacillus*, *Pseudomonas*, *Raoultella, Achromobacter*, *Acidomonas*, *Brevibacterium*, *Pseudomonas*, *Streptomyces*, *Serratia*, *Sphingobium*, *Clostridium, Klebsiella*, and *Lysinibacillus* have been characterized for pyrethroid degradation (Cycoń and Piotrowska-Seget, 2016; Birolli et al., 2019; Hu et al., 2019; Zhao et al., 2019). Fungi also have the potential to degrade wide variety of pesticides (Maqbool et al., 2016). Only a few groups of fungi including *Aspergillus niger*, *Aspergillus terricola*, *Trichoderma viridae*, *Phaenerochaete chrysosporium* (Saikia and Gopal, 2004; Deng et al., 2015), *Candia pelliculosa* (Chen et al., 2012c), and *Cladosporium* sp. (Chen et al., 2011b) have been reported for pyrethroid biodegradation (Birolli et al., 2018). Fungi have been found to possess comparatively better pesticide degradation potential than bacteria (Bhatt, 2019; Gangola et al., 2019). Many researchers have predicted pyrethroid degradation metabolites and pathways. A few metabolites are common among all pyrethroids, which are used as metabolic markers (such as 3-phenoxybenzoic acid) during microbial degradation. Esterase enzymes are often studied for pyrethroid degradation, due to their presence in bacteria, fungi, insect, and human tissues (Liu et al., 2017; Wang et al., 2018; Bai et al., 2019). Different genes with complete open reading frames coding pyrethroid hydrolase/esterase enzymes have been reported in bacterial strains (Hu et al., 2019; Yang et al., 2019).

Previous studies have concluded that microbial cultures can efficiently remove pyrethroids from the environment. In this review, we have attempted to compile the related information about the toxicity and microbial degradation of pyrethroid insecticides.

## Hazardous Effects of Pyrethroids

Toxicity studies have revealed several effects of pyrethroids on human and marine life (Table 2). Large-scale application of pyrethroids affects humans and animals. Indoor pyrethroids exposure studies revealed low levels of pyrethroids absorption in biological and environmental samples (Ghazouani et al., 2019). Measurement of absorbed daily dose (ADD) from biological samples is more reliable than environmental samples (Williams et al., 2003). Cyfluthrin studies on a medium pile of nylon carpet suggested that pyrethroids were absorbed in the surrounding surfaces and were also found in human urine samples (Williams et al., 2003; Sullivan et al., 2019). Presence of 4-fluro-3 phenoxybenzoic acid in urine samples indicated human exposure to pyrethroids and environmental measurements further confirmed the results (Williams et al., 2003). Studies on pyrethroid residues in children diaper revealed that pyrethroid metabolites were stable on the diaper up to 72 h (Hu et al., 2004). Pyrethroid residues have been reported in dust, cloth, union suit samples, diaper, military uniform, and urine samples (Bradman et al., 2007; Proctor et al., 2019). Pyrethroid residues in the urine samples of pregnant women have been reported from Jiangsu China and France (Qi et al., 2012; Dereumeaux et al., 2018; Kamai et al., 2019). Children in United States are more exposed to pyrethroids as compared to organic food taking children of other areas. Pyrethroid metabolite 3-phenoxybenzaldehyde was commonly found in the urine samples of exposed children (Lu et al., 2009; Dalsager et al., 2019). Presence of 3-phenoxybenzaldehyde metabolite in the semen of Japanese males suggested that their semen quality was decreased by pyrethroids (Toshima et al., 2012). Residues of organophosphorous and pyrethroids were also reported in Australian preschool children (Babina et al., 2012) and urinary concentration of pyrethroids from Queensland (Australia) pre-schoolers correlated with the age and sex (Li et al., 2019). Pyrethroids are also used to disinfect the aircrafts and presences of 3-phenoxybenzaldehyde in the urine samples of flight attendants (18–65 years old) clearly indicated pyrethroid exposure in different age groups of humans (Wei et al., 2012). Higher pyrethroids exposure was reported in farmers and consumers of northern Thailand (Hongsibsong et al., 2019) and extensive studies revealed their carcinogenic potential (Navarrete-Meneses and Pérez-Vera, 2019).

**Table 2:** Hazardous effects of different pyrethroids.

S. No.	Pyrethroids	Sample source/Study sample	Specific statement	References
1	Pyrofenofos, cypermethrin, permethrin, tefluthrin, *trans*-fenfluthrin, bifenthrin, indoxacarb, acephate, and spinosyn A	Larvae of tobacco budworm, *Heliothis virescens* (F.)	Detection of esterase resistance/susceptibility in insect larvae	Huang and Ottea, 2004
2	Racemic (*cis*-bifenthrinm, fonofos, and profenofos), racemic *trans*-permethrin, *cis*-permethrin	Freshwater, invertebrates	Determination of enantioselectivity based toxicity of chiral pyrethroids	Liu et al., 2005
3	Cyfluthrin	Human fetal astrocyte cells	Affecting growth, survival and functioning of human astrocyte cells	Mense et al., 2006
4	*Cis* and *trans* permethrin	Human urine	Detected the presence of pyrethroid in urine	Bradman et al., 2007
5	Cyfluthrin	Human peripheral lymphocytes	Genotoxic effect seen in the human peripheral lymphocytes due to mutation	Ila et al., 2008
6	Type I and Type II pyrethroids	Mild poisoning sign	Hyper activity and hyper-excitability in mice and rat	Wolansky and Harrill, 2008; Wang et al., 2018
7	Type I and Type II pyrethroids	Moderate to severe poisoning sign	Prostration, sinuous writhing, uncoordinated twitches, normothermia	Wolansky and Harrill, 2008; Wang et al., 2018
8	Type I and Type II pyetrhoids	Nearly lethal syndrome	Clonic seizures, tonic seizure, and rigors occasionally just before death	Wolansky and Harrill, 2008
9	Cyfluthrin and beta cyfluthrin	Androgen responsive cell line, MDA-kb2	Antiangrogenic activity was reported in presence of pyrethroids	Zhang et al., 2008
10	λ-Cyhalothrin and γ-cylaothrin	Aquatic invertebrate and Fish	Single enantiomer is less toxic than racemate of pyrethroid	Giddings et al., 2009
11	Racemic pyrethroid	Urine sample	Detection of pyrethroid intermediate 3-phenoxybenzoic acid	Lu et al., 2009
12	α-Cypermethrin	Human peripheral blood lymphocytes	High cytotoxic effect at >20 μg/ml	Kocaman and Topaktaş, 2009
13	Bifenthrin, permethrin, fenvalerate	Yeast strains	Enantioselctivity in esterogenic activity	Wang et al., 2010
14	Deltamethrin, β-cyfluthrin, cypermethrin, permethrin, bifenthrin, esfenvalerate, λ-cyhalothrin, tefluthrin, fenpropathrin, resmethrin, and S-bioallethrin	Swiss-webster mice	Pyrethroid actions affect the sodium influx in cerebrocortical neurons	Cao et al., 2011
15	Beta-cypermethrin	Soil samples	Microbial community in soil affected by the action of cypermethrin	Zhuang et al., 2011
16	Cypermethrin and decamthrein	Brinjal fruits	It was noticed that trace quantity persist in Brinjal upto a long time	Kaur et al., 2011
17	Lambda-cyhalothrin	Developing rats	Cholinergic dysfunctions and oxidative stress is responsible for neurotoxicity in rats	Ansari et al., 2012
18	Cypermethrin	*Myrica opima*	Hepatopancreas and gill have increased glycogen	Tendulkar and Kulkarni, 2012
19	Deltamethrin	Human dopaminergic neuroblastoma SH-SY5Y cells	Oxidative stress mediated neurotoxicity	Romero et al., 2012
20	Deltamethrin	Male BALB/c Mice	Deltamethrin inhibit the osteoclast development	Sakamoto et al., 2012
21	Deltamethrin	Rat bone marrow cells	Testicular injury and genotoxicity due to pyrethroids when compare with biopesticide (*Bacillus thuringiensis*)	Ismail and Mohamed, 2012
22	Cypermethrin	CV-1 cells (*Cercopithecus aethiops* monkey kidney Cells)	Cypermethrin inhibited the interaction of androgen receptor and steroid receptor coactivator-1	Pan et al., 2012
23	Deltamethrin	*Trichogramma evanescencs, T. semblidis*	Discrimination of sex pheromones affected by deltamethrin	Delpuech et al., 2012
24	Bifenthrin	Rat adrenal pheochromocytoma cells (PC-12)	Bifenthrin affects the antioxidant enzyme due to enantioselectivity	Lu, 2013
25	3-Phneoxybenzoic acid	Urine and semen samples	Pyrethroid exposure reduced semen quality	Toshima et al., 2012
26	Pyrethroids (3-phenoxybenzoic acid, 2-methyl 3-phenoxybenzoic acid)	Urine samples	Chronic exposure of pyrethroids on Australian preschool childrens	Babina et al., 2012
27	Pyrethroids mixed	Urine samples of flight attendant	Detection of pyrethroid metabolites in urine analysis	Wei et al., 2012
28	Lambda-cyhalothrin	Liver of *Oreochromis niloticus*	Apoptotic and oxidative effect due to piperonyl butoxide treatment with lambda- cypermethrin	Piner and Üner, 2012
29	Permethrin	Mice	Reproductive toxicity due to enatioslectivity of permethrin	Jin et al., 2012
30	Bifenthrin	Sandy loam soil	Difference of half life in sterile and non sterile soil indicated that bifenthrin persistence change microbial community	Sharma and Singh, 2012
31	Twelve different pyrethroids	Marine animals (Dolphins)	Mother to calf transfer of pyrethroids by lactation and gestation in Dolphin	Alonso et al., 2012
32	Thirteen different pyrethroids	Human breast milk	Analysis of pyrethroid in Brazil, Columbia and Spain by food samples to humans than transfer rate in infants	Corcellas et al., 2012
33	Permethrin	Consumed human food, residential exposure	Mathematical modeling of EPA that is SHEDS-multimedia model	Zartarian et al., 2012
34	Lambda-cyhalothrin	Male mice	Reproductive and Hepatotoxicity observed	Al-Sarar et al., 2014
35	Pyrethroids and metabolites	Urinary sample of pregnant women	Data indicated effect of pyrethroid on pregnant women that will also affect infants	Qi et al., 2012
36	Permethrin, cfluthrin, esfenvalerate, cypermethrin	Mammalian cells, fishes	Pyrethroids act as endocrine disruptor	Brander et al., 2016
37	λ-Cyhalothrin, fenvalerate and permethrin	Embryo of Zebra fish (*Danio rerio*)	Triiodothyronine (T3) level decreased due to exposure of lambda cyhalothrin and Fenvalerate	Zhang et al., 2017
38	Cypermethrin, deltamethrin and cyhalothrin	*Cucumis sativus*	Chlorophyll and caretonoids showed sensitive effect	Braganca et al., 2018
39	Cypermethrin	*Bacillus* sp.	*In vitro* toxicity detected in human cell line	Sundaram et al., 2013
40	Bifenthrin, λ-cyhalothrin, cyfluthrin, cypermethrin, *cis*-deltamethrin, esfenvalerate, and *cis/trans* permethrin	Solid food sample	Pyrethroid degradates not present in sufficient level in diet to substantially impact the adults	Birolli et al., 2016b; Morgan et al., 2018
41	Cypermethrin	*Salvator merianae* (Argentine tegu)	Cypermethrin with other pesticides affect immune and endocrine system	Mestre et al., 2019

Macrophages are immune cells that play important role in pathogen removal from the cells. It was observed that β-cypermethrin and cyhalothrin treatment decreased phagocytic activity and nitric oxide production in macrophage cells (He et al., 2019). No activity was detected at low concentration of cyhalothrin whereas macrophage activity was blocked at higher concentration. Direct effect of cyhalothrin on macrophage cells is due to the activity of sodium ion membrane channels whereas activity of hypothalamus pituitary adrenal axis caused indirect effects in rats (Righi and Palermo-Neto, 2005; He et al., 2019). Different levels of pyrethroid toxicity in freshwater invertebrates *Ceriodephnia dubia* and *Daphnia magna* is due to a selective enantiomer in racemate (Liu et al., 2005). Comparative study of cyfluthrin and chlorpyrifos toxicity in human fetal astrocytes (star shaped glial cells in the brain and spinal cord) revealed that cyfluthrin exerts more toxic effects on survival, growth and proper functioning of human peripheral lymphocytes, and induces apoptosis (Mense et al., 2006; Segura et al., 2018). Cyfluthrin and chlorpyrifos over express pro-inflammatory mediators, and cyfluthrin can cause mutation to change chromosome number (Mense et al., 2006; Muzinic et al., 2018). Genotoxic and cytotoxic effects of cyfluthrin were detected by Salmonella/mammalian microsome mutagenicity test, chromosomal aberration, chromatid exchange, and micronucleus formation in cultured human peripheral blood lymphocytes *in vitro* (Ila et al., 2008; Chalap et al., 2018). Pyrethroid genotoxicity demands for their restricted use around children, elderly people, and pregnant women (Kocaman and Topaktaş, 2009).

Pyrethroids are neurotoxic pesticides and affect neurotransmitters (Gammon et al., 2019). Effect of low acute oral dose of pyrethroids has been investigated in small rodents. Neurobehavioral study suggested that pyrethroids block sodium chloride and GABA channels, which inhibit transfer of neurotransmitters between cells (Cao et al., 2011; Richardson et al., 2019). Permethrin was reported against *Laccophilus minutus* (Touylia et al., 2019) and in humans it is absorbed through dermal and non-dietary entry points (Nakagawa et al., 2019). Cypermethrin, allethrin, *cis*/*trans* permethrin and deltamethrin modified the strength and behavior of tested organisms, whereas decreased grip strength was noted after pyrethrum, cypermethrin, bifenthrin, β-cyfluthrin, deltamethrin, S-bioallethrin, and permethrin treatments. A coordination study of deltamethrin and α-cypermethrin with rotarod revealed that the compound with α-cyano group enhanced acoustic evoked startle response amplitude whereas opposite effect was observed without α-cyano group. Intensity of tremor and sensory response is rarely explored against pyrethroids (Wolansky and Harrill, 2008). Ansari et al. (2012) reported that long term exposure of λ-cypermethrin produces harmful neurochemical endpoints that cause behavioral variations in rats.

Antiandrogenic activity of cyfluthrin and β-cyfluthrin in a carcinogenic cell line MDA-kb2 has also been reported (Zhang et al., 2008). Bifenthrin evokes various toxicological effects in different human cells by modifying homeostasis and cell viability in human prostate cancer cells (Chien et al., 2019). Bifenthrin acts as endocrine disrupting chemical by inhibiting the expression of glucocorticoid and estrogen receptor (Ligocki et al., 2019). 5-Dihydrotestosterone induced androgen receptor activity was blocked by pyrethroids in MDA-kb2 cells and considered as moderate antiandrogenic (Zhang et al., 2008). Giddings et al. (2009) compared the effect of γ-cyhalothrin and λ-cyhalothrin, and suggested that the single active enantiomer (isomer) causes more toxicity than racemic mixture of both pyrethroids in marine fish and invertebrates (Giddings et al., 2009). Lambda-cyhalothrin and fenvalerate decreased triiodothyronine (T3) in the embryo of Zebra fish (*Danio rerio*) (Awoyemi et al., 2019). Due to specific binding between ERα receptor and pyrethroid isomer, synthetic pyrethroids act as estrogenic endocrine disrupting compounds (Wang et al., 2010; Lauretta et al., 2019).

Damage of β-cypermethrin to soil microbial communities is less as compared to marine life (Zhuang et al., 2011). Bifenthrin affects microbial community in sandy loam soil and pyrethroids are generally considered as a threat to marine life (Sharma and Singh, 2012). Cypermethrin and deltamethrin residues were reported in Brinjal fruits which can be reduced by washing and boiling before cooking (Kaur et al., 2011). Deltamethrin induced shift of soil microorganisms was reported with cabbage plants after 30 days of treatment (Braganaca et al., 2019). Pyrethroids are highly toxic to aquatic organisms such as fish, shrimp, crab and shellfish. Effects of γ-cyhalothrin and modulator piperonyl butoxide were observed in fish *Oreochromis niloticu*s. Study revealed that λ-cyhalothrin causes oxidative stress in the liver of *O. niloticus* and stress was further increased in the presence of piperonyl butoxide (Piner and Üner, 2012; Giddings et al., 2019). Pyrethroids transform into solid, liquid and gas phase and enter in food chains to pose high health risk. Pyrethroids accumulated in sediment are major source of aquatic toxicity (Tang et al., 2018). Toxicity of type I and type II pyrethroids was assessed in embryo of Zebrafish (*D. rerio*) that depicted different mechanistic effects of pyrethroids and their instability in marine environment (Awoyemi et al., 2019). Pyrethroids toxicity to red blood cells and brain cells is associated with physiological changes and DNA damage in fish (Paravani et al., 2019; Ullah et al., 2019).

Cypermethrin stress decreased total glycogen content in different organs/tissues of *Marica opima* and affected its metabolic activity (Tendulkar and Kulkarni, 2012). Oxidative stress produced by deltamethrin is one of the major mechanism of neurotoxicity (Romero et al., 2012). Deltamethrin inhibits the differentiation of osteoclast by regulating nuclear factor of activated T-cells cytoplasmic-1 (NFATc-1) and oxygenase-1 which is an important regulatory protein (Sakamoto et al., 2012). Deltamethrin is more hazardous than biopesticide (*Bacillus thuringiensis*) and has been reported to cause testicular injury in rats and affect sex pheromones (Delpuech et al., 2012; Ismail and Mohamed, 2012). Cypermethrin inhibits the androgen receptor (AR) activity by disrupting AR-SRC1 (steroid receptor coactivator-1) interaction (Pan et al., 2012). Toxicity of pyrethroids (*cis*-bifenthrin) is enantioselective in nature and particular degrading enzymes are more expressive. These previous studies provide detailed knowledge of chiral chemical toxicity at molecular level (Lu, 2013). High pesticide exposure leads to acute pesticide poisoning and damages central nervous system (CNS) (Starks et al., 2012).

Permethrin and its four chiral isomers caused severe histopathological testicular damage in mice at 100 mg/kg by decreasing testis weight and concentration of testosterone hormone (Jin et al., 2012). These pesticides have been noted to transfer from mother to calf in dolphins via gestation and lactation pathways (Alonso et al., 2012; Kondo et al., 2019). Studies conducted in Brazil, Columbia and Spain reported the presence of pyrethroids in human breast milk at concentrations of about 1.45–24.2 ng/gm lw (Corcellas et al., 2012).

Zartarian et al. (2012) studied the effects of permethrin in 3–5 years old children. Stochastic human exposure and dose stimulation model (SHEDS) for multimedia multi-pathway chemicals is commonly known as multimedia computer based method developed by environmental protection agency (EPA) for the study of toxic chemicals (Zartarian et al., 2012). Lambda-cypermethrin has been reported to cause reproductive toxicity, hepatotoxicity, splenotoxicity, and nephrotoxicity in male mice (Starks et al., 2012). Effect of pyrethroids on different fish suggested that highly lipophilic pyrethroids accumulate in sediments and organisms. These compounds also act as endocrine disruptor and block the hormonal signaling in aquatic animals and mammals (Brander et al., 2016).

A study of cypermethrin, deltamethrin, and cyhalothrin phytotoxicity on *Cucumis sativus* showed that these insecticides affected the production of cholorophyll and caretonoids in plants (Braganca et al., 2018). The study on cypermethrin biodegradation and metabolites detection in tomato, cabbage, rape, pepper, and cucumber revealed its rapid dissipation in plants. Enatioselective degradation was observed in pepper and cucumber (Yao et al., 2018).

## Pyrethroid-Degrading Microorganisms and Their Degradation Characteristics

Many studies have confirmed that bacteria and fungi are capable of degrading pyrethroids in liquid cultures or soils (Table 3). Microorganisms can degrade pyrethroids by using either directly as a source of carbon or co-metabolically (Birolli et al., 2016b; Cycoń and Piotrowska-Seget, 2016; Chen and Zhan, 2019). *Acidomonas* sp. degraded more than 70% of allethrin in 72 h as carbon and nitrogen source (Paingankar et al., 2005). *Micrococcus* sp. strain CPN1 has been reported to biodegrade and completely mineralize cypermethrin through enzymatic cleavage of ester bond (Tallur et al., 2008; Zhao et al., 2015). Pyrethroid degrading bacterium *Sphingobium* sp. JZ-2 was isolated and characterized from activated sludge of pyrethroid manufacturing wastewater. Strain JZ-2 efficiently degraded cypermethrin, bifenthrin, and fenvalerate. Novel pyrethroid hydrolase purified from the cell extract was strongly inhibited by different ions (Ag^+^, Cu^2+^, Hg^2+^, and Zn^2+^) (Guo et al., 2009). *Serratia* spp. strain JC1 and JCN13 efficiently biodegraded beta-cypermethrin due to their higher hydrophobicity. Strain JC1 degraded 92% beta-cypermethrin within 10 days whereas strain JCN13 degraded 89% within 4 days. Growth conditions for better biodegradation were also optimized through response surface methodology (RSM) and Box-Behnken design (Zhang et al., 2010). Pyrethroid degrading bacterium *Raoultella ornithinolytica* ZK4 was isolated from the soil samples of a pesticide plant and it degraded lambda-cyhalothrin and deltamethrin (Zhang et al., 2019). Recently 3-phenoxybenzoic acid and other pyrethroids were degraded (96.37%) within 72 h of treatment by using *Klebsiella pneumoniae* strain BPBA052 (Tang et al., 2019).

**Table 3:** Pyrethroid degrading microorganisms and their optimized conditions in lab/field.

S. No	Bacteria/Fungi/ Insect/Other	Pyrethroid used	Standard condition for growth	Specific statement	References
1	*Bacillus cereus*, *Pseudomonas fluorescence*, and *Achromobacter* sp.	Permethrin, deltamethrin, fastac, fenvalerate, and fluvalinate	pH-7.0 Temp-30°C Tween,80 to maintain relatively insoluble compound in solution	3-Phenoxybenzoic acid was the major product Permethrin transformed rapidly as compared to others	Maloney et al., 1988
2	*Pseudomonas* sp. ET1	3-Phenoxybenzoate	pH-7.2 Temp-30°C	Phenoxy substituted benzyl aldehyde was metabolized whereas benzyl alcohol, benzene, phenol, and aniline were not	Toppw and Akhtar, 1991
3	*Trichoderma viridae*, *Trichoderma terricola*, *Aspergillus niger*, and *Phanerochate chrysosporium*	Beta-cyfluthrin	pH-6.5°C czapek dox medium used	Cleavage of ether linkage result in metabolites formation. That is confirmed by NMR analysis	Saikia and Gopal, 2004; Deng et al., 2015
4	*Acidomonas* sp.	Allethrin	pH-7.0 Temp-37°C with minimal salt medium	Allethrin is metabolized by hydrolytic pathway followed by dehydrogenation and oxidation	Paingankar et al., 2005
5	*Pseudomonas stutzeri* S1	Beta-cyfluthrin	pH-7.0 Temp-28°C Minimal salt media	Strain able to degrade the beta-cyfluthrin	Saikia et al., 2005
6	*Aspergillus niger* ZD11	*Trans*-permethrin, *cis*-permethrin, cypermethrin, fenvalerate, and deltamethrin	pH-6.8 Temp-30°C Minimal salt media	Novel pyrethroid hydrolase having the potential of wide range of pyrethroid degradation	Liang et al., 2005; Deng et al., 2015
7	*Micrococcus* sp. CPN1	Cypermethrin	Seuberts mineral salt medium at 150 rpm	Presence of 3-phenoxybenzoate, protochatachauate, and phenol were investigated	Tallur et al., 2008
8	*Bacillus* sp.	Cypermethrin	pH-7.0 Temp.30°C Rpm-110 Minimal salt medium	3-Phenoxybenzaldegyde and other metabolites of the pathway	Bhatt et al., 2016b, 2019b
9	*Sphingobium* sp. JZ-2	Fenpropathrin, cypermethrin, permethrin, cyhalothrin, deltamethrin, fenvalerate, and bifenthrin	pH-7.0 Temp-30°C Luria Bertani medium	3-Phenoxybenzadihyde, 2,2,3,3-tetramethylcyclopropanecarboxylic acid, 3-phenoxybenzaldehyde, 3-phenoxybenzoate, protocatechuate, and catechol	Guo et al., 2009
10	*Serratia* spp.	Beta-cypermethrin	pH-6-9 Temp-20–38°C	3-Phenoxybenzoic acid, phenol (92% degradation occurs within 10 days by *Serratia* strains)	Zhang et al., 2010
11.	*Ochrobactrum tritici* pyd-1	*Cis* and *trans* permethrin, fenpropathrin	Luria Bertani medium Temp-30°C	2,2,3,3-Tetramethylcyclopropane carboxylic, 3-phenoxybenzaldehyde, 3-phenoxybenzoic acid, 4-hydroxy-3-phenoxybenzoic acid, protocatechuate, and p-hydroquinone	Wang et al., 2011
12	*Clostridium* sp. ZP3	Fenpropathrin	pH-7.5 Temp-35°C	Benzyl alcohol, benzenemathanol, and 3,5-dimethylamphetamine	Zhang S. et al., 2011
13	*Pseudomonas aeruginosa* CH7	Beta-cypermethrin	pH-6-9 Temp-25–35°C	Biosurfactant production increased beta-cypermethrin degradation	Zhang C. et al., 2011
14	Neustonic and epiphytic bacteria	Deltamethrin	pH-7.0 Temp-20°C Minimal salt medium	Bacteria reduced the initial concentration of cypermethrin	Kalwasinska et al., 2011
15	*Bacillus cereus* MTCC1305	Fenvalerate	pH-6-7.4	HPLC analysis showed 500 ppm fenvalerate degradation by the bacterium	Selvam et al., 2013
16	*Pseudomonas viridoflava*	Fenvalerate	pH-6.2-7.0	HPLC analysis showed the pyrethroid is degraded with different peak areas	Selvam et al., 2013
17	*Serratia marcescens*	Deltamethrin	No data	3-Phenoxybenzaldehyde and peaks of other metabolites	Cycoń et al., 2014
18	*Acinetobactor calcoaceticus* Mcm5	Cypermethrin, bifenthrin, cyhalothrin, and deltamethrin	pH-7.0 Temp-30°C	All the pyrethroid degraded by the bacterial strain Mcm5	Akbar et al., 2015b
19	*Azorcus indigens* HZ5	Cypermethrin	pH-7.0 Temp-30°C	70% cypermethrin degradation after 144 h	Burns and Pastoor, 2018
20	*Bacillus* sp. SG2	Cypermethrin	pH-7.0 Temp-32°C	82% cypermethrin degraded after 15 days of experiment	Bhatt et al., 2016b, 2019a
21	*Bacillus* sp. DG-02	Fenpropathrin, cypermethrin, cyfluthrin, lambda-cyhalothrin, deltamethrin, permethrin, and bifenthrin	pH-7.5 Temp-30°C	Different biodegradation patterns followed with distinct concentration	Chen et al., 2012b, 2014
22	*Bacillus amyloliquifaciens* AP01	Cypermethrin	pH-7.0 Temp-30°C	Approximately 45% cypermethrin degradation observed in 5 days	Lee et al., 2016
23	*Bacillus megaterium* Jcm2 *Brevibacillus parabrevis* Jcm4	Cypermethrin, bifenthrin, cyhalothrin, and deltamethrin	pH-7.0 Temp-30°C	Maximum 89% degradation obtained in cypermethrin	Akbar et al., 2015a
24	*Brevibacterium aureum* DG-12	Cyfluthrin, cyhalothrin, fenpropathrin, deltamethrin, bifenthrin, and cypermethrin	pH-7.0 Temp-27°C	Maximum 84.7% biodegradation observed with cyfluthrin	Chen et al., 2013a
25	*Catellibacterium* sp. *CC-5*	Cypermethrin, fenvlerate, fenpropathrin, deltamethrin, permethrin, and cyhalothrin	pH-7.0 Temp-30°C	90% biodegradation achieved after 7 days with cypermethrin and deltamethrin	Zhao et al., 2013
26	*Lysinbacillus sphaericus* FLQ-11-1	Cyfluthrin	pH-7.0 Temp-35°C	Approximately 80% cyfluthrin removal after 5 days	Hu et al., 2014
27	*Ochrobactrum lupini* DG-S-01	Cypermethrin, cyfluthrin, fenpropathrin, cyhalothrin, and deltamethrin	pH-7.0 Temp-30°C	Maximum 90% biodegradation obtained with cypermethrin within 5 days	Chen et al., 2011a
28	*Pseudomonas aeruginosa* JQ-41	Fenpropathrin, cypermethrin, deltamethrin, bifenthrin, and cyhalothrin	pH-7.0 Temp-30°C	Maximum 91.7% biodegradation obtained with fenpropathrin after 7 days of experiment	Song et al., 2015
29	*Pseudomonas flourescens*	Cypermethrin	pH-7.0 Temp-25°C	37.2% cypermethrin degraded in absence of sucrose after 96 h	Grant et al., 2002
30	*Rhodococcus* sp. Jcm5	Cypermethrin, bifenthrin, cyhalothrin, and deltamethrin	pH-7.0 Temp-30°C	100% cypermethrin catabolism occures in 10 days	Akbar et al., 2015b
31	*Stenotrophomonas* sp. ZS-S-01	Fenvalerate, deltamethrin, cypermethrin, cyfluthrin, and cyhalothrin	pH-7.0 Temp-30°C	Catabolic degradation in case of fenvalerate complete degradation occurs in 6 days	Chen et al., 2011d
32	*Streptomyces* sp. HU-S-01	Cypermethrin	pH-7.5 Temp-26–28°C	90% cypermethrin degradation in 24 h	Lin et al., 2011
33	*Streptomyces aureus* HP-S-01	Cypermethrin, deltamethrin, cyfluthrin, bifenthrin, fenvalerate, fenpropathrin, and permethrin	pH-7.5-7.8 Temp-27–28°C	Cyfluthrin, bifenthrin and fenvalerate degraded completely within 5 days	Chen et al., 2011c, 2012d
34	*Candia pelliculosa* ZS-02	Bifenthrin, cyfluthrin, deltamethrin, fenvalerate, cypermethrin, and fenpropathrin	pH-7.2 Temp-32°C	Only bifenthrin degraded completely within 5 days	Chen et al., 2012c
35. clc	*Cladosporium* sp. HU	Fenvalerate, fenpropathrin, cypermethrin, deltamethrin, bifenthrin, and permethrin	pH-7.2 Temp-26°C	Fenvalerate, fenpropathrin, cypermethrin degraded completely within 5 days	Chen et al., 2011b
36	*Phaenerochate chrysosporium*	Cyfluthrin	pH-6.5 Temp-28°C	Co-metabolic degradation (60%) after 30 days of experiment	Saikia and Gopal, 2004
37	*Bacillus cereus* BCC01	Beta-cypermethrin, deltamethrin, cypermethrin, permethrin, fenvalerate, and cyhalothrin	pH-7.0 Temp-30°C	Six metabolites were detected after biodegradation: α-hydroxy-3-phenoxy-benzeneacetonitrile, 3-phenoxybenzaldehyde, methyl-3-phenoxybenzoate, 3,5-dihydroxybenzoic acid, 3,4-dihydroxybenzoic acid, and 3,5-dimethoxyphenol	Hu et al., 2019
38	*Bacillus subtilis* BSF01	Cypermethrin, deltamethrin, cyhalothrin, and β-cyfluthrin	pH-6.7 Temp-34.5°C	*Cis*/*trans* β-cypermethrin, 3-(2,2-dichloroethenyl)-2,2-dimethylcyclopropanecarboxylate, α-hydroxy-3-phenoxybenzeneacetonitrile, 3-phenoxybenzaldehyde, 3-pheoxybenzoic acid, and 3,5-dimethoxyphenol	Xiao et al., 2015
39	*Pseudomonas fulva* P31	D-phenothrin	pH-7.3 Temp-29.5°C	3-Phenoxybenzaldegyde and 1,2- benzene dicarboxylic butyl dacyl ester identified as major intermediates	Yang et al., 2018
40	*Sepedonium maheswarium*	Beta-cyfluthrin	PDA media Temp-25 ± 2°C	Dissipation study	Mukherjee and Mittal, 2007
41	*Penicillum raistrickii* CBMAI 93*, Aspergillus sydowii* CBMAI935*, Cladosporium* sp. CBMAI 1237*, Microsphaeropsis* sp. CBMAI1675*, Acremonium* sp. CBMAI 1676*, Westerdykella* sp. CBMAI 1679, and *Cladosporium* sp. CBMAI1678	Esfenvalerate	pH-7 Temp-32°C	All fungal strains degraded esfenvalerate with different efficiencies	Birolli et al., 2016a; Zhao et al., 2016
42	*Aspergillus* sp. CBMAI 1829*, Acremonium* sp. CBMAI 1676*, Microsphaeropsis* sp. CBMAI 1675, and *Westerdykella* sp. CBMAI 1679	Lambda-cyhalothrin	pH-7 Temp-32°C	Enantioselctive degradation of cyhalothrin by the fungal strains	Birolli et al., 2016a; Zhao et al., 2018
43	*Cunninghamella elegans* DSM1908	Cyhalothrin	pH-5.6 Temp-28°C	Intermediate metabolites and proposed pathways identified	Birolli et al., 2018
44	*Phtobacterium genghwense* PGS6046	Cyfluthrin	pH-8 Temp-30°C	Characterized metabolites in different culture conditions	Wang et al., 2018
45	*Acinetobacter baumanii* ZH-14	Permethrin	pH-7.0 Temp-30°C	Strain degraded permethrin as well as wide variety of pyrethroids	Zhan et al., 2018
46	*Raoultella ornithinolytica* ZK4	Pyrethroids	pH-6.5, Temp-37°C	Bacteria was isolated from the soil sediment that degraded different pyrethroids	Zhang et al., 2019
47	*Klebsiella pneumoniae* BPBA052	3-Phenoxybenzoic acid	pH-7.7 Temp-35.01°C	Bacterium uses 3-phenoxybenzoic acid as carbon and energy source	Tang et al., 2019
48	*Rhodopseudomonas* sp. PSB07-21	Fenpropathrin	pH-7.0 Temp-35°C	Photoheterotrophic mode of growth was better as compared to photoautotrophic growth mode	Luo et al., 2019

Aerobic and anaerobic soil biodegradation of pyrethroid etofenprox was investigated in the rice fields of California. 3-Phenoxybenzoic acid, a hydrolytic product of ester bond cleavage was not detected in any sample. Microbial population in a flooded soil (anaerobic) played role in conversion and dissipation of etofenprox (Vasquez et al., 2011; Furihata et al., 2019). β-Cyfluthrin is commonly used by Indian farmers at Indian Agriculture Research Institute Delhi for controlling *Lepidepteran* pests of *Solanaceous* crops. To degrade its soil residues, *Pseudomonas stutzeri* was isolated, and identified using enrichment culture technique and intermediate metabolites were confirmed according to the previous reported pathway (Saikia et al., 2005; Birolli et al., 2019). Cyfluthrin degradation by bacterium *Photobacterium ganghwense* was confirmed by comparative metabolomics (Wang et al., 2019). Pyrethroid degradation capability of *Ochrobacterium tritici* strain pyd1 is dependent upon the molecular structure of synthetic pyrethroids (Wang et al., 2011). Strain pyd-1 effectively degraded both, *cis* and *trans* isomers at the same rate. Detailed metabolic pathway of fenpropathrin biodegradation through strain pyd-1 was also identified. Specific enzyme activities of pyrethroid hydrolase, 3-phenoxybenzaldehyde (PBD) dehydrogenase, 3-phenoxybenzoic acid (PBA) hydroxylase, 4-hydroxy-PBA dioxygenase, and p-hydroquinone hydroxylase have been studied in relation to pyrethroid fenpropethrin (Dehmel et al., 1995; Wang et al., 2011; Luo et al., 2019). *Pseudomonas pseudoalcaligenes* strain POB310 has been reported for the degradation of 3- and 4-carboxydiphenyl ethers (Dehmel et al., 1995). A genetically engineered strain of *Pseudomonas putida* also degraded other pesticides similar to pyrethroids (Gong et al., 2018). Anaerobic bacterium *Clostridium* strain ZP3 isolated from the mixed wastewater and sludge samples degraded higher concentrations of fenpropathrin by co-metabolic activity and was used to analyze complex redox reaction in fenpropathrin biodegradation (Zhang S. et al., 2011; Zhao et al., 2016). Co-metabolic biodegradation of β-cypermethrin was explored with *Bacillus licheniformis* B-1 (Zhao et al., 2019). *Pseudomonas aeruginosa* CH7 degraded 90% of beta-cypermethrin by isomerization within 12 days. Bio-surfactant (rhamnolipid) promotes the adsorption and hydrophobicity of chemical compounds (Zhang C. et al., 2011). Neustonic and epiphytic bacteria and their mixed cultures were noted to similarly, degrade deltamethrin (Kalwasinska et al., 2011). Chen et al. (2012d) also validated the cypermethrin biodegradation through *Bacillus cereus* ZH-3 and *S. aureus* HP-S-01 cells.

*Ochrobactrum anthropi* strain YZ-1 is quite potent to degrade pyrethroids. Role of bacterial esterase PytZ (606 bp) in biodegradation without any cofactor has been confirmed (Zhai et al., 2012). Pyrethroid hydrolase of molecular weight 53 KDa was purified and characterized from *A. niger* strain ZD11. Pyrethroid activity was not detected in the presence of glucose and it indicates that pyrethroid hydrolase only expresses in fungus after pyrethroid stress. Optimum pH for *A. niger* was found to be lower than *B. cereus* (7.3) whereas the optimum temperature was comparatively higher (45°C) as compared to *B. cereus* strain SM3 (37°C). Enzyme activity inhibition by thiol modifying enzyme (PCMB) p-chloromercuribenzoate suggested that sufhydryl group was involved in the catalytic center of enzyme (Liang et al., 2005). Cypermethrin reportedly caused toxicity to human hepatocarcinoma cell line H4H7 (Sundaram et al., 2013). *Bacillus* sp. helps to biodegrade cypermethrin in soil microcosm and *B. cereus* MTCC 1305 has been reported to biodegrade fenvalerate (Selvam et al., 2013). Pyrethroid toxicity and biodegradation efficiency of *Pseudomonas viridoflava* has also been thoroughly investigated (Selvam et al., 2013; Thatheyus and Selvam, 2013). Two strains of *S. marcescens* DeI-1 and DeI-2, enhanced the disappearance of cypermethrin (Cycoń et al., 2014). Application of ammonium nitrate as external nitrogen at the rate of 122.1 kg/ha^-1^ increased cypermethrin degradation by 80% (Xie et al., 2008). External nitrogen might accelerate microbial metabolism in lag phase.

Metabolic and ecological potential of fungi makes them suitable for bioremediation and waste treatment (Harms et al., 2011). Cell free extracts of fungi are known to effectively degrade chlorpyrifos and pyrethroids (Yu et al., 2006). β-Cyhalothrin degradation by different fungi has been reported including *Trichoderma viridae* strain 5-2, *Trichoderma viridae* strain 2211, *Phanerochaete chryosogenum*, *Aspergillus terricola*, and *A. niger*. Study was followed by the extraction and identification of major degradation metabolites (Saikia et al., 2005; Birolli et al., 2019).

Radiolabeled (^14^C) permethrin was used to understand the mechanism of pyrethroid degradation in soil and sediment. It was observed that R-enantiomer of both *trans* and *cis* permethrin mineralized rapidly as compared to S-enantiomer and degradation product of *cis* permethrin was more persistent in the soil environment (Qin and Gan, 2006). Enantioselective degradation of pyrethroids was also performed at southern California under field condition (soil and sediment) and enantioselective degradation of *cis*-bifenthrin, cypermethrin and permethrin occurred at half-life of 270–277 days, 52–135 days, and 99–141 days, respectively. Absence of enantioselectivity in biodegradation represents preferential condition for transformation (Qin et al., 2006).

Axenic culture of *Pseudomonas fluorescens*, *B. cereus*, and *Achromobacter* sp. degraded different pyrethroids such as permethrin, fenvalerate, fastac, deltamethrin, and fluvalinate in the presence of Tween-80 and 3-phenoxybenzoic acid was the major metabolite. Permethrin rapidly transformed into 3-phenoxybenzoic acid as compared to other pyrethroids (less than 5 days). In soils, pyrethroids were degraded into a diaryl ether metabolite 3-phenoxybenzoate. Efficiency of *Pseudomonas* strain ET1 in 3-phenoxybenzoate metabolism per cell was calculated as 2.6 ± 0.9 × 10^-13^ gm/cell/hour. Strain *Pseudomonas* ET1 morphologically resembles with *Pseudomonas delafieldii* but differs in 3-phenoxybenzoate degradation (Toppw and Akhtar, 1991). One strain cannot degrade all aromatic compounds due to the structural differences except genetically modified strains, which can be modified to simultaneously degrade different compounds (Gong et al., 2018).

## Gene Cloning and Enzymatic Characterization of Pyrethroid Carboxylesterases

Esterease (carboxyl ester hydrolase) play an important role in initial transformation of parent pyrethroid by attacking ester bond or cytochrome P-450 dependent monooxygenase on acid or alcohol moieties (Kamita et al., 2016). Many researchers have studied carboxylesterase isolation and purification from *B. cereus* SM3, *Klebsiella* sp. ZD112, *Sphingobium* sp. JZ2, *Pseudomonas* flourescens SM-3, *A. niger* ZD11, *Ochrobactrum lupini* DG-S-01, *Streptomyces aureus* HP-S-01, *Streptomyces* sp. HU-S-01, *Pseudomonas stutzeri*, *Micrococcus* sp. CPN 1, *Serratia* sp. JC1 and *Serratia* sp. JCN13, *Pichia pastoris* (Cycoń and Piotrowska-Seget, 2016; Liu et al., 2017; Tang et al., 2017). Limitations in culture dependent approaches are popularizing the metagenomics tools. Thermostable pyrethroid esterase Sys410 was investigated by metagenomic approach and enzyme contained 280 amino acids having a molecular mass of 30.8 KDa (Fan et al., 2012; Popovic et al., 2017). Cloning was carried out from metagenomic library of soil samples and sequence analysis revealed that 819 bp *pye3* gene codes for 273 amino acid protein. Enzyme was further characterized on the basis of enzyme kinetics (*K*_m_ and *K*_cat_ activity) (Li et al., 2008; Luo et al., 2018).

Reported pyrethroid hydrolases have different pH and molecular weight. Carboxyl esterase enzymes can catabolize wide array of similar ester containing compounds. Because of enantioselectivity, a few essterases exhibit specific or moderate kinetic abilities, which differ from pyrethroid degrading enzymes. Enzyme expression and metabolites production during pyrethroid degradation is differential and can be sequentially up-regulated or down regulated (Bhatt et al., 2019b). Metagenomic based library was useful for the identification and mining of pyrethroid degrading genes, such as *pytY* and *pytZ* (*O. anthropi* strain YZ1), *estP* (*Klebsiella* sp. JD112), *pytH* (*Sphingobium* sp. JZ-1), and *pye* (soil). These genes can be used for isolation and comparison of novel pyrethroid degrading microbial strains.

Bacterial cells produce CO_2_ from 3-phenoxybenzoate at *K*_m_ (Michaelis constant) value of 1.4 ± 0.8 μM that reveals high affinity of bacterial cells to 3-phenoxybenzoate. Metabolism of this pyrethroid intermediate is constitutive rather than catabolite repression. Maloney et al. (1993) were the first to report enzymatic catalysis of pyrethroids in *B. cereus* strain SM3. Enzyme initially named as permethrinase (61 ± 3 KDa) was finally termed as carboxylesterase after successive studies. Pure culture and cell free extract of *B. cereus* SM3 successfully hydrolyzed 2nd and 3rd generation pyrethroids. Permethrin was hydrolyzed more rapidly as compared to flumethrin.

Esterase is ranked under subcategory of hydrolases and International Union of Biochemistry classified carboxylesterase as subgroup 3.1.1. Active site of this enzyme contains serine residue that plays role in acylation during pyrethroid biodegradation through nucleophilic attack by hydroxyl group (OH). Transformed pyrethroid metabolites are easily excreted in urine because of their better water solubility than original pyrethroids. It justifies high concentrations of carboxylesterase enzyme in mammalian serum and liver (Sogorb and Vilanova, 2002). There are two major categories of carboxylestearases in human body (carbocylesterase-1 and carboxylesterase-2), which can degrade pyrethroids (Wang et al., 2018). Pyrethroid *trans* forms are more easily degraded by carboxylesterases as compared to *cis*-isomer. Due to high affinity for Na^+^ channels, *trans* isomers are more toxic to mammalian tissues. Rabbit serum contains higher cypermethrin degradation activity (WHO Task Group on Environmental Health Criteria for Permethrin et al., 1990).

Novel pyrethroid hydrolyzing esterase was reported from *Klebsiella* sp. strain ZD112. Gene *estP* contains an open reading frame of 1914 bp, encoding a protein of 637 amino acids and molecular mass of 73 KDa. Purified enzyme can effectively degrade wide variety of ester bond containing pesticides. *K*_m_ value for *trans* and *cis* permethrin indicated that *EstP* has higher catalytic power than carboxylesterase enzyme (Wu et al., 2006). A novel *pytH* esterase gene, coding pyrethroid hydrolyzing carboxylesterase was also reported in *Sphingobium* sp. strain JZ1 having an open reading frame of 840 bp. Further cloning and purification of this enzyme revealed its molecular weight of about 31 KDa, isoelectric point (pI) of 4.85, and it does not require any cofactor for degrading different pyrethroids (Wang et al., 2009). Degradation of fenpropathrin and fenvalerate in alkaline and acidic soil was observed as enantioselective under aerobic conditions (Li et al., 2009).

Co-expression of two target genes [organophosphate hydrolase (*opd*) and carboxylesterase B1 (*b1*)] from *Falovobacterium* sp. and *Culex pipens* is used for degradation of organophosphorous, carbamate, and pyrethroid pesticides. Carboxylesterase 001D that was isolated from *Helicoverpa armigera* and heterologously expressed in bacteria (*E. coli*) potentially hydrolysed cypermethrin and fenvalerate (Li et al., 2016). Advanced genetic engineering techniques can enable a single microorganism to degrade multiple pesticides (Lan et al., 2006).

Fungal enzymes have also been reported for pesticide biodegradation. Some fungal enzymes catalyze esterification, hydroxylation, dehydrogenation, and deoxygenation during the degradation process. *A. niger* YAT carries out etherification reaction during cypermethrin biodegradation. Similar to metabolites of pyrethroid bacterial biodegradation, degrading enzymes of fungal strains have been confirmed as well (Maqbool et al., 2016).

Carboxylesterases of *Lucila cuprina* and *Drosophila melanogaster* with mutagenesis in active site were used to study pyrethroid degradation. Carboxylesterase was cloned and expressed by genetic engineering to observe their pyrethroid degradation efficiency (Heidari et al., 2005). Human liver carboxylesterase *hcE1* and *hcE2* degraded both type I and type II pyrethroids with stereoselcetivity. *trans*-isomers were degraded more rapidly by these enzymes as compared to *cis*-isomer (Table 4). *K*_m_ values of enzyme catalysis were lower as compared to pyrethroid compounds (Nishi et al., 2006). Human, rat and rabbit hepatocarboxylesterases also depicted capability to degrade pyrethroids (Ross et al., 2006). Esterases in *Heliothis virescens* larvae were found to be associated with their resistance to pyrethroids (Huang and Ottea, 2004). Development of next generation sequencing (NGS) methods has enabled us to use genetically engineered microorganism for large-scale pyrethroid hydrolases. Heterologous expression of human and insect pyrethroid hydrolases can be more beneficial for pyrethroid removal from contaminated sites.

**Table 4:** Pyrethroid degrading enzymes from different sources.

S. No.	Pyrethroid isomer used for study	Microbes/other source	Enzymes	Metabolites	References
1	*Trans*-permethrin, *cis*-permethrin, and racemic permethrin	*Bacillus cereus*	Permethrinase with molecular weight 61KDa	B-naphthylacetate was used as substrate, no specific data of pathway reported	Maloney et al., 1993
2	*Trans*-permethrin and *cis*-permethrin	*Lucila cuprina* and *Drosophila melanogaster Helicoverpa armigera*	Carboxylestease enzyme plays role in pyrethroid hydrolysis	No data	Heidari et al., 2005; Li et al., 2019
3	*Trans*-permethrin, *cis*-permethrin, cypermethrin, fenvalerate, and deltamethrin	*Aspergillus niger* ZD11	Novel pyrethroid hydrolase degrades permethrin and similar compounds	No data	Liang et al., 2005
4	Deltamethrin, bifenthrin, cyfluthrin, and λ-cyhalothrin	Human liver	hCE-1 and hCE-2 carboxylesterases hydrolyze the pyrethroids and pyrethroid like fluorescent surrogates	No data	Nishi et al., 2006; Wang et al., 2018
5	Pyrethroids and organophosphate	*Falobacterium* sp., *Culex pipiens*	Co-expression of organophosphate hydrolase and carboxylesterase B1 gene that can degrade many pesticides together	No data	Lan et al., 2006
6	*Trans* and *cis*-permethrin	*Klebsiella* sp. ZD112	Esterase enzyme with molecular weight 73KDa has high efficiency than insect and mammals	p-Nitrophenyl ester was used for enzyme catalysis	Wu et al., 2006
7	Permethrin, deltamethrin, cypermethrin, and esfenvalerate	Intestinal, liver and serum carboxylesterse	Hydrolysis of pyrethroids by humans and rat tissues	No data of metabolite	Crow et al., 2007
8	Bioresmethrin α-cypermethrin deltamethrin	Hepatic cells	Hepatic carboxylesterase	Hepatic carboxylesterase metaboloize ester comtaining xenobiotics	Ross et al., 2006
9	Cypermethrin	Soil samples	Soil dehydrogenase	Increased dehydrogenase activity when nitrogen was added into cypermethrin	Xie et al., 2008
10	Prethroids in soil	Soil samples	Pyrethroid hydrolyzing esterase	The genes coding esterase cloned and expressed from metagenomic library	Li et al., 2008
11	Cypermethrin	*Bacillus* spp.	Esterase and aldehyde dehydrogenase	Upregulation of the enzymes in response to pesticide stress	Bhatt et al., 2019a
12	Cypermethrin	*Bacillus* sp.	Esterase, dehydrogenease, and many other proteins and enzymes	Differential expression was observed with cypermethrin in *Bacillus* sp.	Bhatt et al., 2016a
13	Permethrin, fenpropathrin, cypermethrin, deltamethrin, cyhalothrin, fenvalerate, and bifenthrin	*Sphingobium* sp. JZ-1	Pyrethroid hydrolyzing carboxylesterase	840bp of gene coding for the enzyme carboxylesterase (molecular mass-31 KDa and PI-4.85)	Wang et al., 2009
14	Fenpropathrin, cypermethrin, permethrin, cyhalothrin, deltamethrin, fenvalerate, and bifenthrin	*Sphingobium* sp. JZ-2	Pyrethroid hydrolase	This enzyme was a monomer of a 31KDa with pI-4.85.	Guo et al., 2009
15	Cyhalothrin, cypermethrin, and deltamethrin	Soil samples	Thermostable pyrethroid esterase	Isolated and identified from metagenomic approach. Molecular mass of the enzyme was 30.8 KDa	Fan et al., 2012
16	Lambda-cyhalothrin, beta-cypermethrin, beta cyfluthrin, deltamethrin, and permethrin	*Ochrobactrum anthropi* YZ-1	Novel pyrethroid hydrolyzing carboxylesterase	Hingh enzyme specificity, broad substrate activity makes this enzyme as a potential candidate for pyrethroid degradation	Zhai et al., 2012
17	Beta-cypermethrin, deltamethrin, cypermethrin, permethrin, fenvalerate, and Cyhalothrin	*Bacillus cereus* BCC01	Carboxylesterase EstA	Enzyme showed excellent adaptability under various circumstances	Hu et al., 2019
18	Fenpropathrin	*Rhodopseudomonas palustris* PSB-S	Esterase (Est3385)	The optimal temperature (35°C) and pH (6.0) for esterase	Luo et al., 2018, 2019
19	Cypermethrin	*Bacillus subtilis*	Esterase and laccase	pH-7.0 Temp-32°C	Gangola et al., 2018

## Metabolic Pathways of Pyrethroid Biodegradation

**Figure 2:**
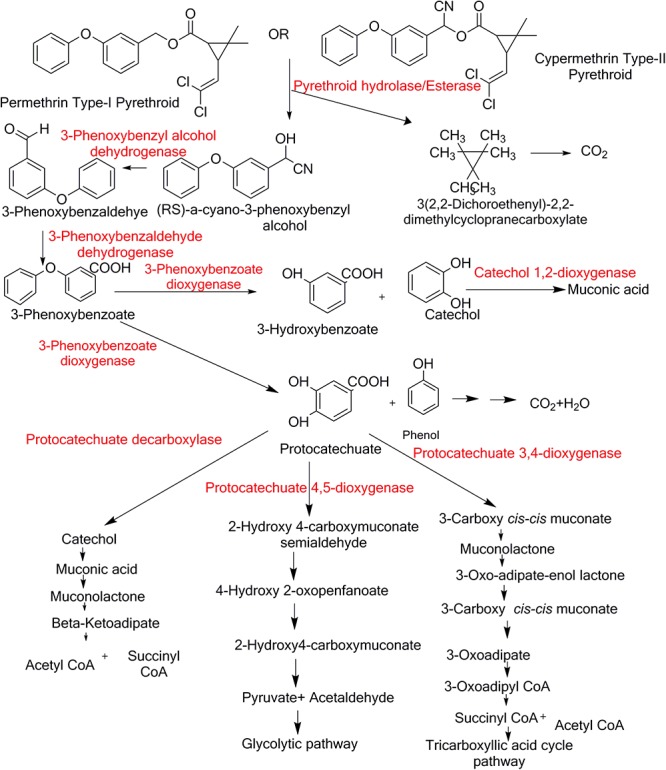
Detailed metabolic pathways of pyrethoids in microorganisms.

Every living cell that can survive in different environmental conditions must has metabolic pathways, which help to fetch required food (nutrition) from the surroundings (soil, water). Oxygenases (monooxygenases and dioxygensases) play important role in biodegradation of pesticides by common pathways (Fuchs et al., 2011; Birolli et al., 2016b; Bhatt et al., 2019b). Pyrethroid degrading cells (bacteria, fungi and some animal cells) produce metabolites and make them accessory for downstream pathways (Figure 2). Casida identified the pyrethroid breakdown pathway in 1960. Pyrethroids are metabolized in human body via catabolic pathway. Distribution of carboxylesterases in different tissues has been reported and major esterase of intestine is called carboxylesterase 2 (hCE2) that has higher catalytic activity as compared to liver and other tissue cells (Crow et al., 2007).

Hydroxyester metabolites are produced during oxidative pathways whereas oxidative ester cleavage is the minor pathway of some pyrethroids. Isomers of various pyrethroids are affected differently by initial hydrolytic attack. Pyrethroid degradation by hydroxyl group (OH^-^) nucleophilic attack under alkaline conditions is similar to simple aliphatic ester. Chemically, ester or nitrile hydrolysis occurs under alkaline conditions. Ester (bond) hydrolysis produces acid (RCOO^-^) and 3-phenoxybenzaldehyde, via fast decomposition of intermediary compound cyanohydrins. Another parallel hydrolysis pathway produces primary amide that again hydrolyze into RCOO^-^ acid and 3-phenoxybenzaldehyde (Wang et al., 2018). Microbial degradation follows the same pattern and most of the metabolites are common in all microbial pathways with only a few exceptions. Strains belonging to genera *Bacillus*, *Micrococcus*, *Staphylococcus aureus*, *R. ornithinolytica*, and *Catellibacterium* are used for pyrethroid detection (Tallur et al., 2008; Chen et al., 2013b; Zhao et al., 2013; Zhang et al., 2019). Consortium biodegradation pathways of *B.cereus* ZH3 and *S. aureus*, and *Bacillus licheniformis* B1 and *Sphingomonas* sp. SC-1 have also been reported (Chen et al., 2012b; Liu et al., 2013; Wang et al., 2019). 3-Phenoxybenzaldehyde and 2,2,3,3 tetramethylcyclopropanecarboxylic acid were detected during cypermethrin degradation by *Bacillus* sp. SG2 and *Bacillus subtilis* BSF01 (Tallur et al., 2008; Bhatt et al., 2016b). Cyclopropanecarboxylic acid, 2,2-dimethyl-3 (2-methyl-1- propenyl), 2-ethyl, 1,3 dimethyl cyclopent 2-ene carboxylic acid, chrysanthemic acid and allethrolone (2-cyclopenten-1-one-4 hydroxy-3 methyl 2 (2 propenyl) were found as major metabolites of allethrin during degradation by *Acidomonas* sp. Hydrolysis, oxidation and dehydrogenation reactions mediated allethrin biodegradation (Paingankar et al., 2005; Bhatt et al., 2016b; Birolli et al., 2016b).

*Bacillus* sp. DG-02 primarily degraded fenpropathrin through carboxylester linkage cleavage to yield 2,2,3,3-tetramethylcyclopropanecarboxylic acid phenyl ester and α-hyroxy-3-phenoxybenzeneacetonitrile which transformed into 3-phenoxybenzaldehyde spontaneously, followed by the oxidization of 3-phenoxybenzaldehyde via diaryl cleavage (Chen et al., 2014). *B. thuringiensis* ZS-19 transformed cyhalothrin by cleavage of both the ester linkage and diaryl bond to yield six intermediate products including α-hydroxy-3-phenoxy-benzeneacetonitrile, 3-phenoxyphenyl acetonitrile, *N*-(2-isoproxy-phenyl)-4-phenoxy-benzamide, 3-phenoxybenzaldehyde, 3-phenoxybenzoate, and phenol, respectively (Chen et al., 2015; Wang et al., 2018). Esterase is essential for ester bond cleavage during pyrethroid degradation. Initially carboxylesterase activity forms two metabolites (*RS*)-α-cyano-3-phenoxybenzyl alcohol and 2,2,3,3 tetramethylcyclopropanecarboxylic acid. Finally 2,2,3,3 tetramethylcyclopropanecarboxylic acid is converted into CO_2_ after few steps but (RS)-α-cyano-3-phenoxybenzyl alcohol is transformed into stable 3-phenoxybenzaldehyde (3-PBA). This step is catalyzed by 3-phenoxybenzaldehyde alcohol dehydrogenase that transroms 3-phenoxybenzaldehyde to 3-phenoxybenzoate. Another enzyme phenoxybenzoate 1,2-dioxygenase transforms 3-phenoxybenzoate into 3,4-dihydroxy- benzoate (protocatechuate) and phenol. Protocatechuate and phenols are further converted into primary and secondary metabolites by microorganisms (Wang et al., 2019). Tricarboxylic acid and glycolysis pathways are mainly used by microbes to produce energy from the pyrethroids (Wang et al., 2014; Gajendiran and Abraham, 2018).

## Recent Tools for Pyrethroid Biodegradation

Traditional identification of microorganisms was based on biochemical tests. Inaccurate results of these tests usually resulted in wrong isolation and characterization. Development of molecular biology tools have facilitated the isolation and identification of pyrethroid degrading microbes (16S rRNA for bacteria and ITS sequencing for fungi) (Gangola et al., 2018; Gupta et al., 2018). Degradation of pyrethroids is commonly analyzed by chromatographic techniques such as high performance liquid chromatography (HPLC), gas chromatography (GC), and mass spectroscopy (MS) (Castellarnau et al., 2016). Due to their low pyrethroid detection limit in soil samples, combination of solid phase extraction and gas chromatography mass spectrometry (GC-MS) was developed as a new method (Chen et al., 2012a; Braganca et al., 2018). After microbial degradation, these methods can efficiently detect pyrethroid metabolites up to ng/gm of soil (Braganca et al., 2018). RSM is generally used for the optimization of pyrethroids and different kinetics of pesticides have been reported (Chen et al., 2012e; Bhatt et al., 2016a,b; Morales et al., 2019). First order reaction is followed for pyrethroid degradation and for the impact analysis on humans whereas cell culture techniques are employed for pyrethroid toxicity detection. Development of rapid genomic tools could analyze the whole genome of pyrethroid catabolizing microorganisms (Bhatt, 2018; Bhatt and Barh, 2018).

## Conclusion and Further Aspects

To feed the world’s rapidly growing population, large-scale use of pesticides in agricultural systems cannot be stopped. Pyrethroid insecticides are used in most of the countries and exhibit comparatively less toxicity than organophosphate and organochlorine pesticides. Recently, toxicity of pyrethroids on marine life (fish), humans and phytotoxicity has been reported. Esterase can degrade ester bond of pyrethroids to produce metabolite 3-penoxybenzaldehyde. Pyrethroid degrading esterase and 3-phenoxybenzaldehyde can be used as signature molecule for pyrethroid biodegradation. Based on this potential marker, pyrethroids degrading microorganism can be selected in a shorter period. Molecular chronometer based coverage of esterase enzyme is possible with existing data. Consortium based pesticide biodegradation approach is more suitable but it has not been significantly studied for pyrethroid degradation. Previous data favors the development of pyrethroid degradation mechanism through microbial system. In future, omics technologies could potentially be used for pyrethroid degradation and to understand molecular biology, enzyme kinetics, and metabolic pathways. System biology of pyrethroid degradation can be further useful for the investigation of multiple information at one platform.

## Author Contributions

SC conceived the idea. PB wrote the manuscript and prepared the figures and tables. YH, HZ, and SC revised the manuscript. All authors approved the final manuscript for publication.

## Conflict of Interest Statement

The authors declare that the research was conducted in the absence of any commercial or financial relationships that could be construed as a potential conflict of interest.
